# Leveraging a disulfidptosis‑related lncRNAs signature for predicting the prognosis and immunotherapy of glioma

**DOI:** 10.1186/s12935-023-03147-7

**Published:** 2023-12-08

**Authors:** Di Chen, Qiaoqiao Li, Yuan Xu, Yanfei Wei, Jianguo Li, Xuqiang Zhu, Hongjiang Li, Yan Lu, Xianzhi Liu, Dongming Yan

**Affiliations:** 1 Department of Neurosurgery, The First Affiliated Hospital of Zhengzhou University, 450052 Zhengzhou, Henan China; 2 Department of Cardiology, The Second Affiliated Hospital of Chongqing Medical University, No. 76 Linjiang Road, 400010 Chongqing, China; 3 The First Clinical Medical College, Gannan Medical University, Ganzhou, 341000 Jiangxi, China

**Keywords:** Disulfidptosis, Long noncoding RNA, Prognostic signature, Glioma, Immune microenvironment, Drug sensitivity

## Abstract

**Background:**

Gliomas, a prevalent form of primary brain tumors, are linked with a high mortality rate and unfavorable prognoses. Disulfidptosis, an innovative form of programmed cell death, has received scant attention concerning disulfidptosis-related lncRNAs (DRLs). The objective of this investigation was to ascertain a prognostic signature utilizing DRLs to forecast the prognosis and treatment targets of glioma patients.

**Methods:**

RNA-seq data were procured from The Cancer Genome Atlas database. Disulfidptosis-related genes were compiled from prior research. An analysis of multivariate Cox regression and the least absolute selection operator was used to construct a risk model using six DRLs. The risk signature’s performance was evaluated via Kaplan-Meier survival curves and receiver operating characteristic curves. Additionally, functional analysis was carried out using GO, KEGG, and single-sample GSEA to investigate the biological functions and immune infiltration. The research also evaluated tumor mutational burden, therapeutic drug sensitivity, and consensus cluster analysis. Reverse transcription quantitative PCR was conducted to validate the expression level of DRLs.

**Results:**

A prognostic signature comprising six DRLs was developed to predict the prognosis of glioma patients. High-risk patients had significantly shorter overall survival than low-risk patients. The robustness of the risk model was validated by receiver operating characteristic curves and subgroup survival analysis. Risk model was used independently as a prognostic indicator for the glioma patients. Notably, the low-risk patients displayed a substantial decrease in the immune checkpoints, the proportion of immune cells, ESTIMATE and immune score. IC50 values from the different risk groups allowed us to discern three drugs for the treatment of glioma patients. Lastly, the potential clinical significance of six DRLs was determined.

**Conclusions:**

A novel six DRLs signature was developed to predict prognosis and may provide valuable insights for patients with glioma seeking novel immunotherapy and targeted therapy.

**Supplementary Information:**

The online version contains supplementary material available at 10.1186/s12935-023-03147-7.

## Background

Glioma, a prevalent type of primary malignant tumor in the central nervous system, constitutes around 80% of all malignant brain tumors and is the foremost cause of death among brain tumor patients [1]. While surgical resection remains the conventional treatment for glioma, it is a challenging task to eradicate it completely due to genetic and epigenetic variations, resulting in its highly invasive and infiltrative nature [2]. Hence, postoperative chemotherapy and radiotherapy have been utilized as adjuvants. Unfortunately, only 0.05–4.7% of glioblastoma patients survive for five years [2, 3]. While recent years have seen a deepening of our comprehension regarding the underlying mechanisms and associated signaling pathways, as well as the genetic risk factors contributing to glioma, the precise pathogenesis of the majority of diffuse gliomas remains enigmatic [4, 5]. This underscores the urgent requirement for the development of a reliable and valid risk model capable of precise diagnosis, tailored treatment, and accurate prognosis estimation for glioma patients.

Disulfidptosis, a newly identified form of cell death, was first reported by BY Gan’s laboratory in 2023 [6]. Unlike other established modes of cell death, such as necroptosis [7], pyroptosis [8], and cuproptosis [9], disulfidptosis is triggered by the aberrant accumulation of disulfides, resulting in disulfide stress and ultimately cell death [6]. Disulfide stress is a condition where reactive oxygen species or other oxidizing agents disturb the normal redox equilibrium of cells by inducing the formation of disulfide bonds between cysteine residues of proteins [10]. Additionally, Liu et al. provided evidence suggesting that actin cytoskeletal vulnerability to disulfide stress produces disulfidptosis and proposed a therapeutic approach targeting disulfidptosis for the treatment of cancer [6]. Therefore, regulating cancer cells’ susceptibility to disulfidptosis could be a promising research avenue in cancer treatment. However, it remains unclear whether disulfidptosis plays a crucial role in glioma, and its relationship with patient survival has not been explored.

Long non-coding RNAs (lncRNAs) have been ascribed with regulatory functions on genome activity, protein modification, and posttranscriptional regulation [11–13]. LncRNAs are increasingly being implicated in the progression and development of gliomas [14]. Moreover, the identification of lncRNA signatures has emerged as a promising tool for prognostic assessment in glioma patients, offering a new clinical framework for precise and personalized treatment of glioma [15]. For instance, Chen et al. have successfully established a cuproptosis-related lncRNAs signature to predict prognosis in glioma [9]. Nevertheless, the mechanisms that underlie disulfidptosis-related long noncoding RNAs (DRLs) in glioma patients are still unknown.

In this study, we intend to establish a DRLs signature in glioma patients, predict overall survival (OS), and investigate the immune landscape and potential drugs through bioinformatic analyses. Subsequently, we employed a multi-faceted evaluative framework that included the concordance index, calibration curves, and receiver operating characteristic (ROC) curves, calculating their respective areas under the curve (AUC) to rigorously assess the model’s discriminative capacity and predictive accuracy. Overall, our study will offer fresh perspectives of DRLs in glioma, which may provide novel insights into the diagnosis, treatment, and prognosis of glioma patients in the future.

## Methods

### Collecting related data and detecting prognostic DRLs

The study cohort consisted of transcriptomic data acquired from glioma patients, encompassing both low-grade glioma and glioblastoma, obtained from TCGA (https://portal.gdc.cancer.gov/). The dataset was last accessed on April 12, 2023. Ten disulfidoptosis-related genes (DRGs) were identified based on prior publications (Table S1) [6, 16], and a network was constructed using Cytoscape software to visualize the interactions between these DRGs and lncRNAs [17]. To determine the correlation between the expression levels of the 10 DRGs and lncRNAs, we performed a Pearson correlation analysis (|Pearson correlation coefficient| > 0.4 and p < 0.001). A multivariate Cox regression analysis and least absolute selection operator were then used to identify prognostic lncRNAs associated with overall survival (OS) in glioma patients [18].

### Development and validation of a prognostic DRLs signature

We randomly divided the 701 glioma samples into a training and a testing set using R package “caret” [19]. The DRLs signature was generated using the training set, while the testing set was employed to validate the signatures. To calculate the risk score for each sample, the values of [Exp (lncRNA) x coef (lncRNA)] were added together, where Exp (lncRNA) represented the expression level of every selected lncRNA and coef (lncRNA) represented its corresponding regression coefficient [20]. Based on the risk scores (with the median risk score used as a cutoff), all the glioma samples were separated into the low- and high-risk groups. Glioma prognoses were evaluated by using K-M curves based on age, gender, grade, and risk scores [21].

### Nomogram design and evaluation

We devised a nomogram to prognosticate patient’s survival outcome in terms of OS for 1, 3, and 5-year periods in the TCGA databases (including the train and test cohorts). We accomplished this using the “survival” and “regplot” R packages [22]. Additionally, to determine the accuracy of prediction at specific time points, we conducted time-dependent ROC curves and calculated their corresponding AUC from OS, utilizing the survival ROC package [23]. Predictive accuracy of the nomogram models was assessed using a calibration curve [24].

### PCA, GO and KEGG analyses

We then conducted a three-dimensional PCA (principal component analysis) to illustrate the spatial arrangement of different risk groups according to the gene matrix derived from all genes, DRGs, DRLs, and risk model [25]. Afterward, we detected genes with differential expression (DEGs) (|log2fold-change (FC)| >1 and adjusted p < 0.05). Furthermore, the DEGs were analyzed for GO and KEGG pathways [26].

### Immune landscape assessment

The immune landscape was evaluated by comparing immune checkpoint expression among different groups, and our results were presented as box plots. To determine the immune infiltration status, we calculated the immune infiltration profiles and used various algorithms, including XCELL, TIMER, QULANTISEQ, MCPCOUNTER, EPIC, CIBERSORT-ABS, and CIBERSORT, to identify differences in the immune response [27]. Single-sample GSEA was used to distinguish immune cells that infiltrated tumors for two risk groups [28]. Our results were further analyzed with ESTIMATE for each sample to obtain stromal scores, ESTIMATE scores, and immune scores [29].

### Analysis of tumor mutational burden (TMB) and IC50 of therapeutic drugs

Using the “maftools” package [30], we conducted an analysis of the TMB and identified significant differences between the high- and low-risk groups. In addition, we used the half-maximal inhibitory concentration (IC50) to predict the sensitivity of gliomas to some immune-related drugs using R packages that included “pRRophetic,” “limma,” “ggpubr,” and “ggplot2” with p < 0.0001 [31]. Spearman’s correlation analysis was also performed to evaluate the correlation between each drug and the risk score.

### Analysis of consensus clusters

We employed consensus clustering analysis to detect potential molecular subgroups that could respond to immunotherapy, based on the expression of prognostic DRLs [32]. The molecular subtypes were identified using the cumulative distribution function approach based on these prognostic DRLs. Next, we examined whether the two clusters had different survival probabilities and proportions of immune cell infiltration.

### Assessing the expression levels of 6 DRLs and evaluating their potential clinical significance in gliomas

We sought to determine the expression levels of 6 DRLs used to establish a prognostic signature in glioma. Furthermore, K-M survival analysis and log-rank comparison of groups were performed to assess their prognostic significance [33]. As well, we performed ROC curve analysis using the AUC value to determine the accuracy of 6 DRLs for glioma.

### Tissue samples collection and RT-qPCR

We collected glioma tissue samples and normal brain tissues from 12 patients undergoing surgical resection between June 2022 and March 2023 at the First Affiliated Hospital of Zhengzhou University, Henan, China. The samples were immediately frozen in liquid nitrogen after tissue resection and mRNA expression level was measured using quantitative reverse transcription polymerase chain reaction (RT-qPCR). The total RNA of the above tissue samples was extracted utilizing the TRIzol reagent (Invitrogen), adhering to the manufacturer’s protocol and our prior study [34]. Subsequently, the data were normalized to glyceraldehyde-3-phosphate dehydrogenase (GAPDH) mRNA expression and quantified employing the 2^−ΔΔCT^ method. Table S2 lists the exact sequences of the PCR primers.

### Statistical analysis

Statistical analyses were performed with R (version 4.1.3) and GraphPad Prism (8.0.2). The Chi-square test is a statistical method used to compare the distribution of categorical variables between two or more groups. In this case, the test was used to compare the distribution of categorical variables between the high- and low-risk groups in the study. To examine the relationship between immune infiltration levels and risk scores, pearson correlations were used, with a p value of 0.05 considered statistically significant.

## Results

### Identifying prognostic DRLs in glioma patients

**Fig. 1:**
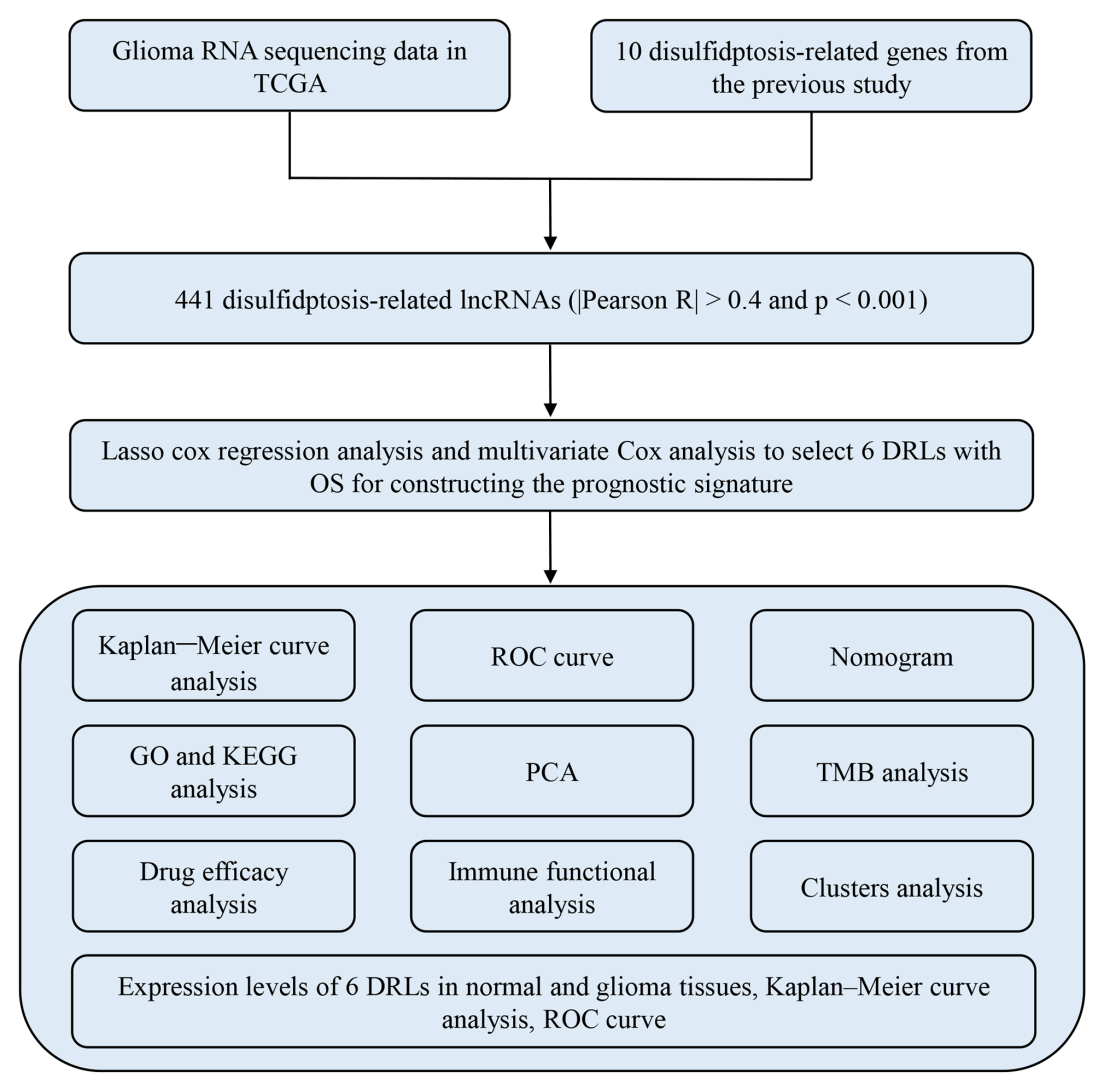
Flow chart of the study design

In this study, we followed a flow diagram as depicted in Fig. 1 to identify prognostic DRLs in glioma patients. Our initial screening focused on 16,876 lncRNAs obtained from the TCGA glioma database. Subsequently, we identified 671 DRLs that displayed a significant correlation with 10 DRGs. Further, we applied Lasso and multivariate Cox regression analyses to identify differentially-regulated genes that exhibited prognostic relevance.

### Building and validating prognostic DRL signatures

**Fig. 2:**
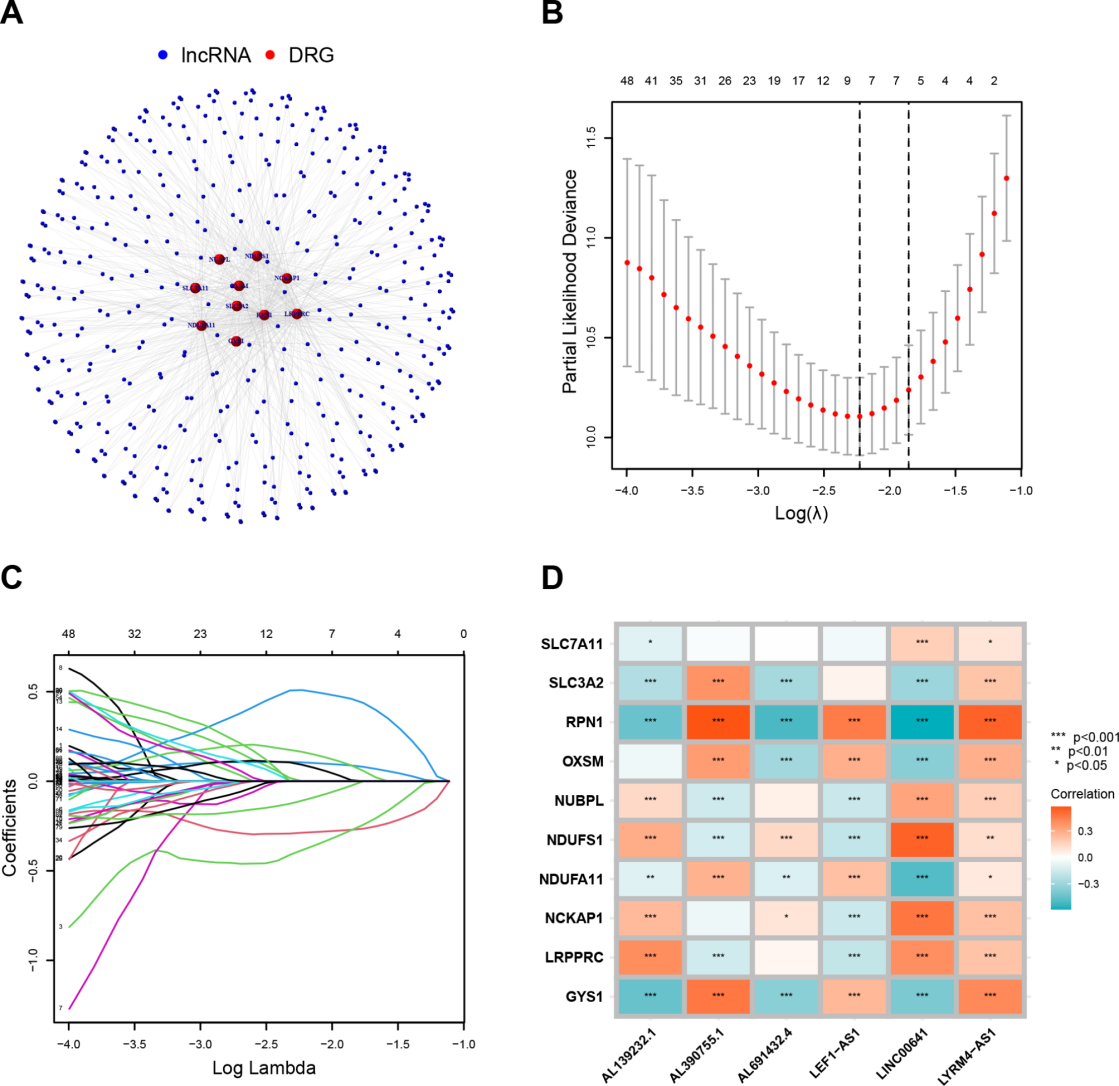
Construction of DRLs prognostic signature. (**A**). The network between disulfidoptosis-related genes (DRGs) and disulfidoptosis-related lincRNAs (DRLs). (**B**). Cross-validation plot for the penalty term. (**C**). Diagram for LASSO expression coefficients. (**D**). Heatmap showing the relationship between DRGs and 6 DRLs. Red indicates positive correlation, while blue indicates negative correlation

102 DRLs were significantly correlated with overall survival in the univariate analysis. Out of these, 43 DRLs were considered as unfavorable prognostic factors for glioma patients (the absolute of hazard ratio HR > 1) (Fig. S1A). A detailed illustration of the relationship between DRGs and DRLs was presented in the Sankey diagram and network (Fig. S1B and Fig. 2A). To avoid overfitting, LASSO regression analysis was performed on the DRLs, and as a result, six DRLs (LINC00641, AL139232.1, AL390755.1, LEF1-AS1, LYRM4-AS1, and AL691432.4) were screened through multivariate Cox regression analysis to build the OS prognostic signature (Fig. 2B-C). There was a correlation between these six DRLs and DRGs on the heatmap (Fig. 2D).

### Distinguishing survival outcomes between high and low-risk groups

**Fig. 3:**
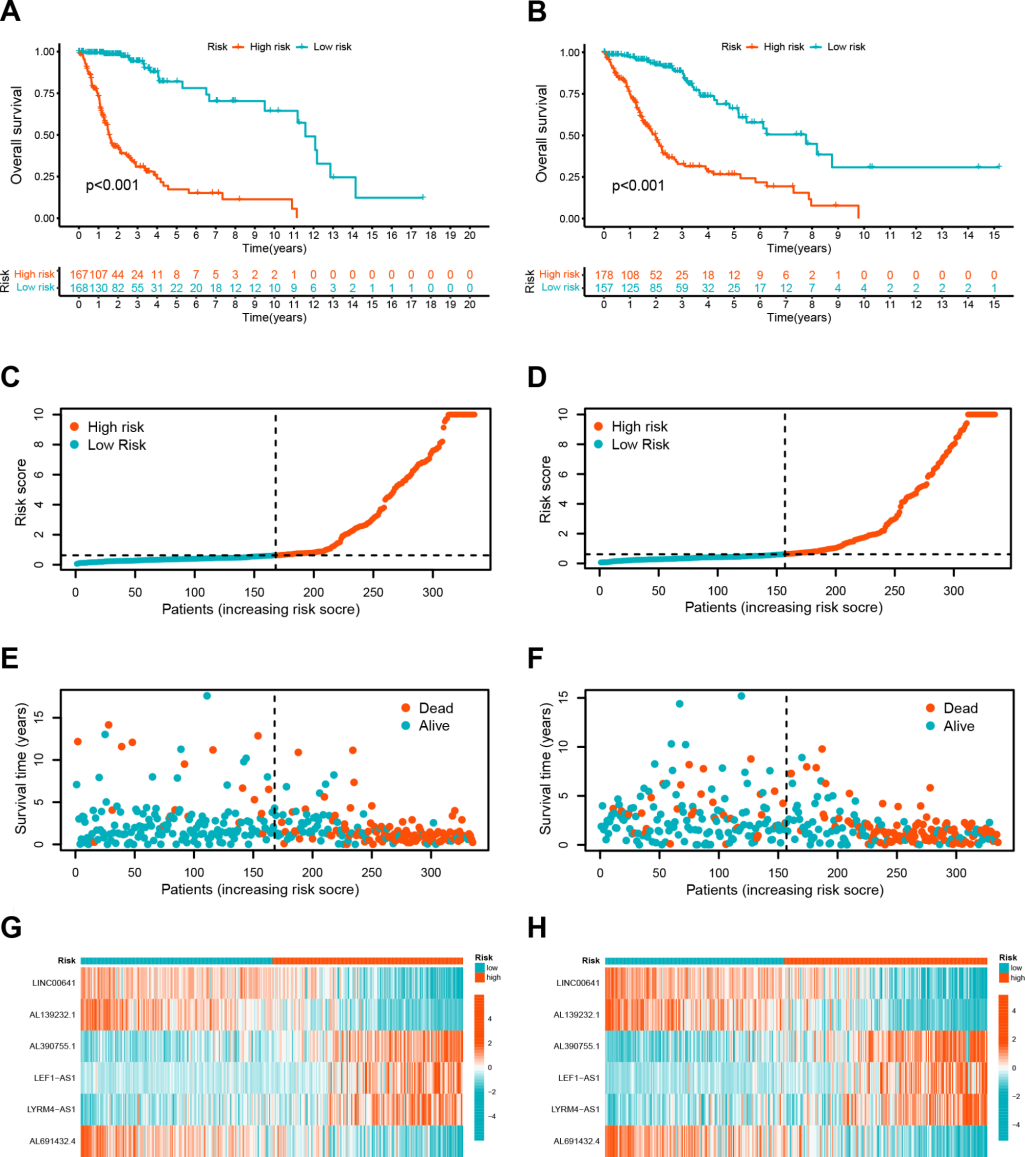
The validation of the prognostic signature of DRLs. (**A**-**B**). Kaplan-Meier curves for overall survival (OS) in the training set (A) and testing set (**B**). (**C**-**D**). Distribution of the DRL-model-based risk score for the training set (**C**) and testing set (**D**). (**E**-**F**). Patterns of survival time and survival status ranked by risk score in the training set (**E**) and testing set (**F**). (**G**-**H**). Heatmap displaying the expression levels of six lncRNAs for glioma patients in the training set (**G**) and testing set (**H**)

Calculation of risk scores is based on the following formula: Risk scores = - (0.339676419092046 × LINC00641 expression) - (0.74150602802086 × AL139232.1 expression) + (0.167235204017436 × AL390755.1 expression) + (0.347174567353386 × LEF1-AS1 expression) + (0.709687113729932 × LYRM4-AS1 expression) - (0.343853790300805 × AL691432.4 expression). The Kaplan-Meier curves illustrated those patients belonging to the low-risk group exhibited significantly longer overall survival in comparison to those in the high-risk group (Fig. 3A-B). A higher risk score also led to a higher mortality rate (Fig. 3C-F). Moreover, a heatmap analysis revealed higher expression levels of three DRLs (AL390755.1, LEF1-AS1, and LYRM4-AS1) in the high-risk group, while the expression levels of three other DRLs (LINC00641, AL139232.1, AL691432.4) were reversed between different risk groups (Fig. 3G-H).

### Assessing the association between DRLs model and clinical characteristics

**Fig. 4:**
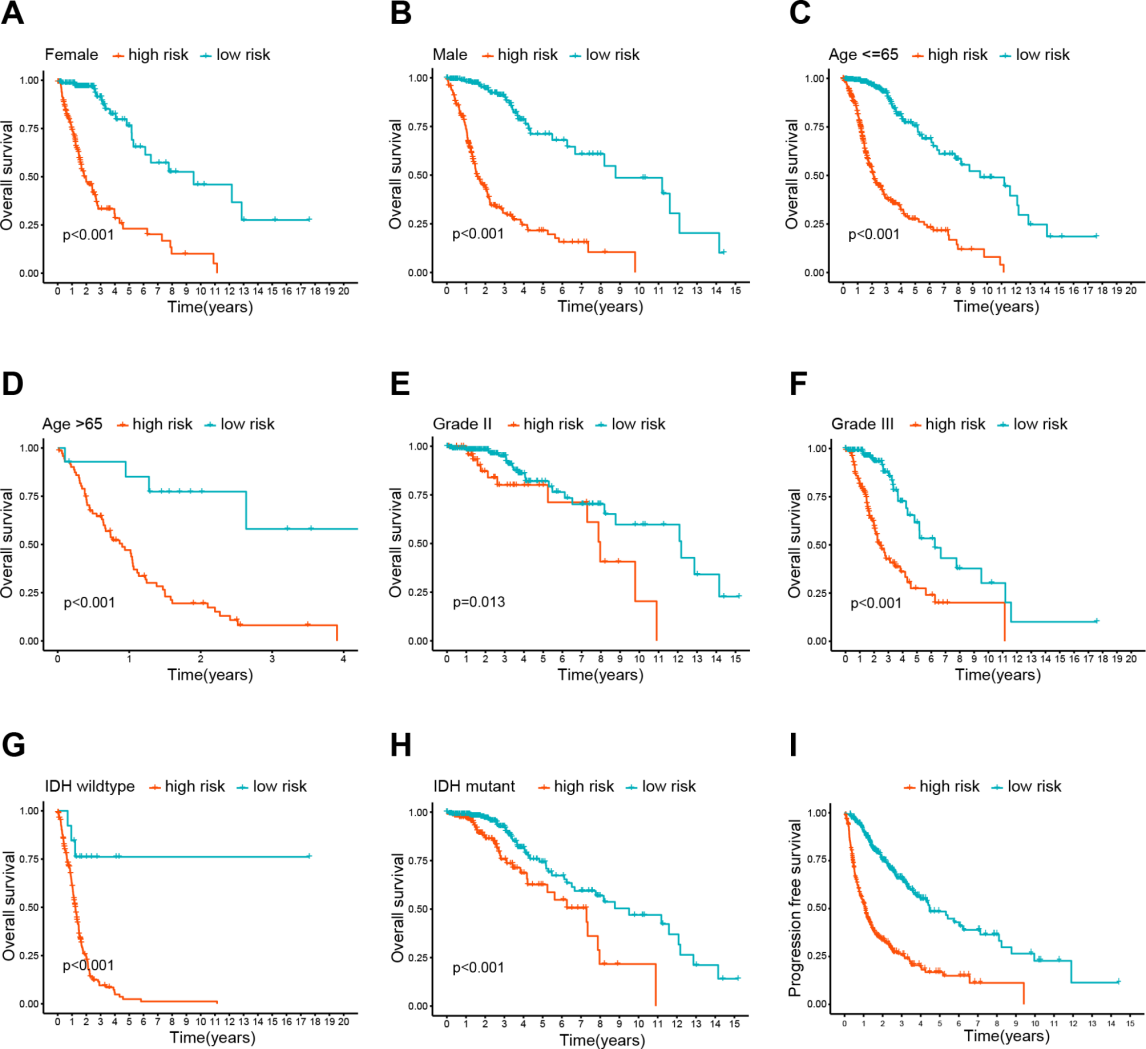
Correlation between the prognostic DRLs signature and clinicopathological features. (**A**-**H**). Kaplan–Meier curve for overall survival in different clinical features such as gender (**A**, **B**), age (**C**, **D**), tumor grade (**E**, **F**) and isocitrate dehydrogenase (IDH) status (**G**, **H**). (**I**). Kaplan–Meier curves for progression-free survival (PFS) in the high- and low-risk groups

Using gender, age, grade, and isocitrate dehydrogenase (IDH) status as subgroups, we examined the association between OS and risk scores. The results revealed that individuals with a high-risk profile exhibited unfavorable outcomes in specific subpopulations, including gender, age, grade, and IDH status (Fig. 4A-H). Furthermore, we evaluated progression-free survival (PFS) for high-risk and low-risk individuals in the TCGA cohort. The results suggested that patients classified into the low-risk group exhibited a more favorable prognosis as opposed to those categorized into the high-risk group (Fig. 4I).

### Development and assessment of the predictive model

**Fig. 5:**
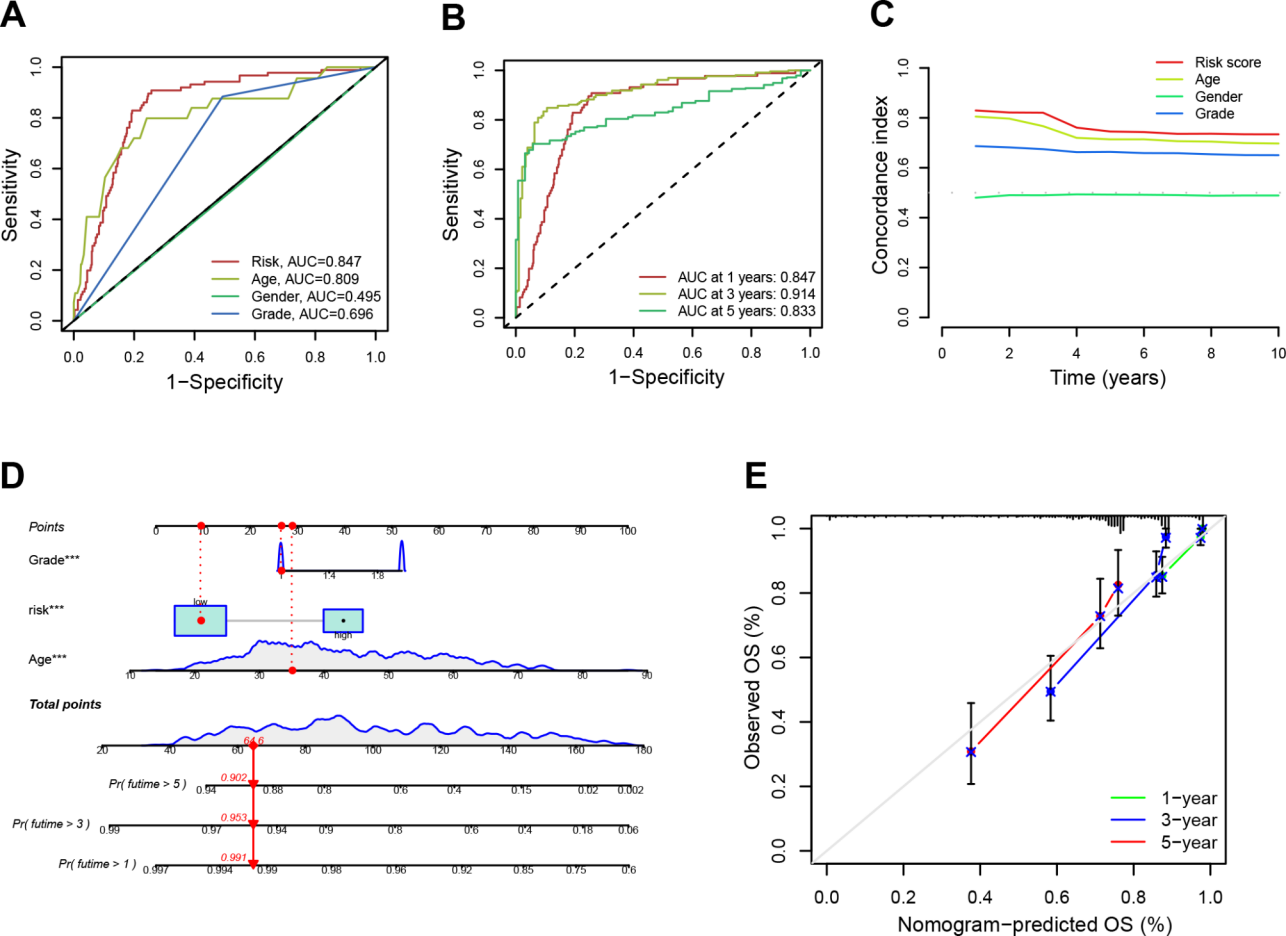
ROC curves and the establishment of a nomogram. (**A**). The plot showing the AUC values for various risk factors, including risk scores, age, gender, and tumor grade. (**B**). The AUC of the CRL signature for 1-, 3-, and 5-year survival rates in glioma. (**C**). The plot showing the concordance index of the risk factors, which includes risk scores, age, gender, and tumor grade. (**D**). A nomogram predicting the 1-, 3- and 5-year survival rates for glioma using independent prognostic factors (grade, age, and risk score). (**E**) A calibration curves indicating the concordance between the prediction by the nomogram and actual survival rates

The potential clinical importance of the DRLs model, along with age, gender, grade, and risk scores, in relation to OS was evaluated (Fig. S2A-B). The findings suggested that the risk model was capable of accurately predicting OS in glioma patients. Moreover, multivariate Cox regression supports the above results (Fig. S2B). Subsequently, ROC curve analysis revealed that the risk score (0.847) had higher AUC than age (0.809), gender (0.495), and grade (0.696) (Fig. 5A). Besides, forecasting the 1-, 3-, and 5-year survival of patients was accurate to 0.847, 0.914, and 0.833 (Fig. 5B). Compared with other clinical characteristics (such as age, sex, and grade), a high risk score provided a better assessment of risk (Fig. 5C). Also, the nomogram predicted OS rates of 0.991, 0.953, and 0.902 at 1, 3, and 5 years (Fig. 5D). Furthermore, a strong correlation was also evidenced between the predicted and observed survival rates, as indicated by the calibration curves (Fig. 5E).

**Fig. 6:**
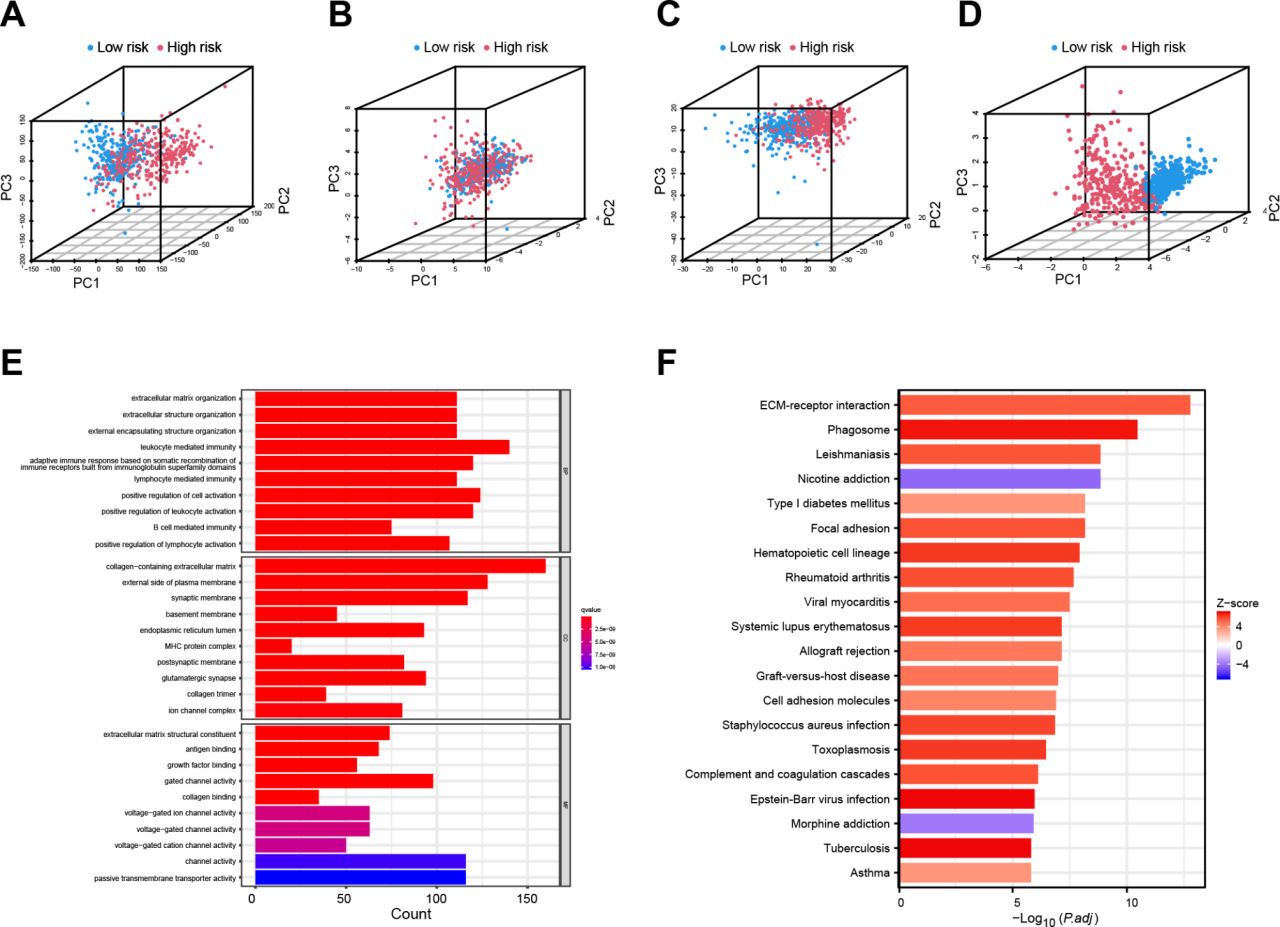
PCA and functional enrichment analysis. (**A**-**D**). The 3D PCA showing the distribution differences between the high- and low-risk groups according to the entire gene expression (**A**), DRGs (**B**), DRLs (**C**), and the risk model genes (**D**). (**E**). GO functional enrichment analysis with a histogram plot (BP, biological process; CC, cellular component; MF, molecular function). (**F**) KEGG pathway enrichment analysis with a histogram plot

### Investigation of PCA and functional enrichment analyses

Using 3-dimensional PCA, we assessed the discriminative power of the DRLs risk signature in distinguishing between low- and high-risk clusters. We found that all genes, DRGs, DRLs, and the 6 DRLs risk signature, with a particular emphasis on the latter, exhibited a remarkable ability to differentiate between the two patient groups (Fig. 6A-D). Additionally, we conducted differential gene expression analysis between the two risk score groups and employed the resulting genes to conduct enrichment analyses. Notably, GO analysis revealed significant enrichment in biological processes related to extracellular matrix organization, adaptive immune response, and leukocyte-mediated immunity (Fig. 6E). Concurrently, the KEGG functional analysis identified several pathways including ECM-receptor interaction, phagosome, nicotine addiction, leishmaniasis, focal adhesion, and Type I diabetes mellitus (Fig. 6F).

### Identification of the immune microenvironment’s association with the prognostic DRL model

**Fig. 7:**
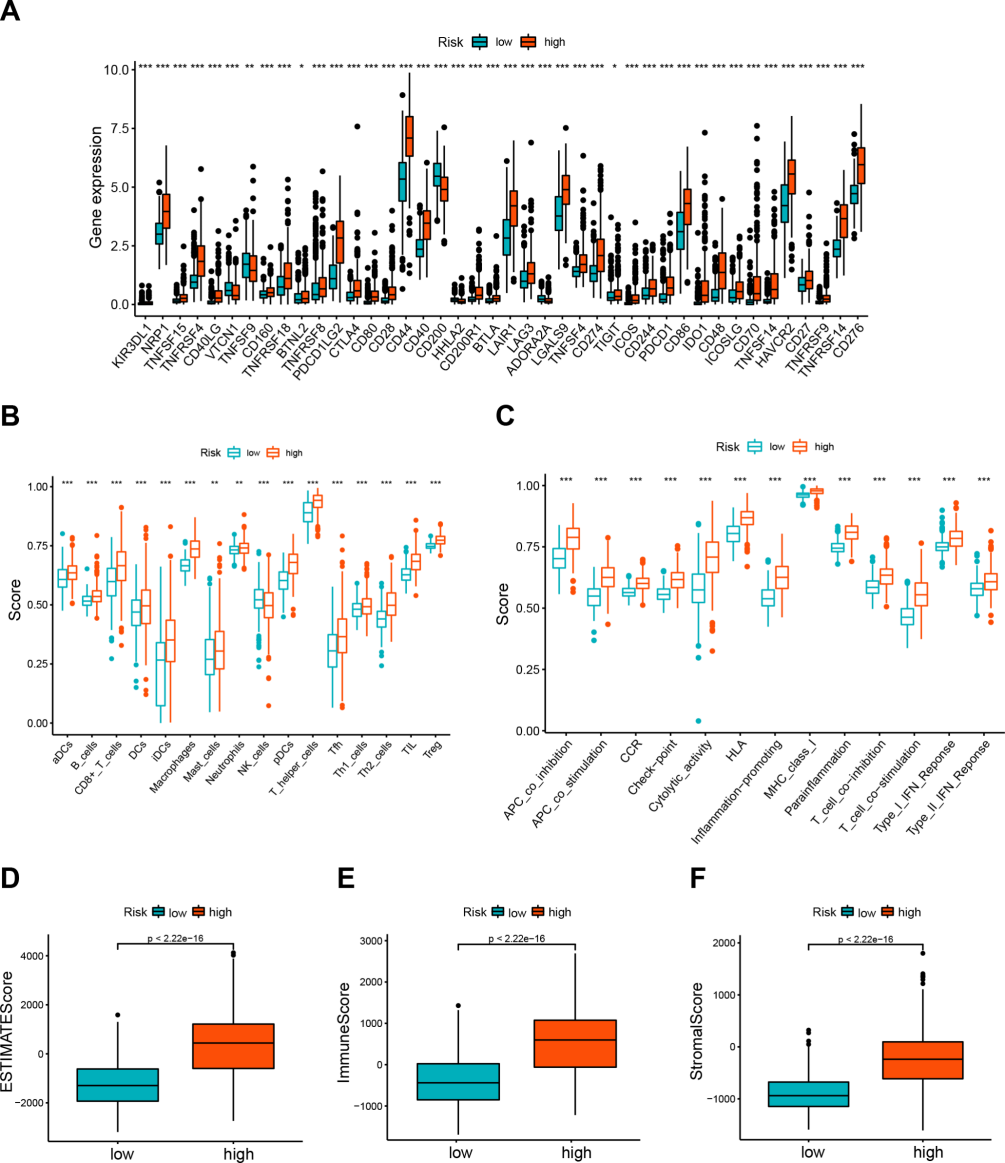
immune microenvironment of DRLs prognostic model. (**A**). The box plot exhibiting the expression level of immune checkpoint genes between the high- and low-risk groups. (**B**). The single-sample gene set enrichment analysis (ssGSEA) displaying the extent of immune cell infiltrations in the high- and low-risk groups. (**C**). The ssGSEA analysis indicating the functions of immune cell subpopulations between the high- and low-risk groups. (**D**-**F**). The ESTIMATE score, immune score, and stromal score in the high- and low-risk groups. *p < 0.05, **p < 0.01, ***p < 0.001

High-risk individuals exhibited a significant increase in immune checkpoint expression level, as indicated in Fig. 7A. The proportion of immune cells was significantly higher among patients with a high-risk signature, except for NK cells (Fig. 7B). Further, high-risk groups had higher Type I and Type II IFN responses that promote inflammation (Fig. 7C). Additionally, the two groups had distinct immune characteristics, as depicted in Fig. 7D-F. Specifically, lower scores were obtained for low-risk patients’ ESTIMATE, immune, and stroma scores. Furthermore, we employed seven distinct algorithms to assess the correlation coefficient of immune cells in the high-risk and low-risk groups, using dot-plots to highlight the differences between these two groups. The results revealed that several immune cells, including B cells, T cell CD4+, T cell CD8+, macrophages, and NK cells, exhibited substantial correlations with risk scores. A heatmap was employed to display the variations in immune function for different risk cohorts (Fig. S3B).

### TMB analysis and evaluation of therapeutic drug sensitivity in glioma

**Fig. 8:**
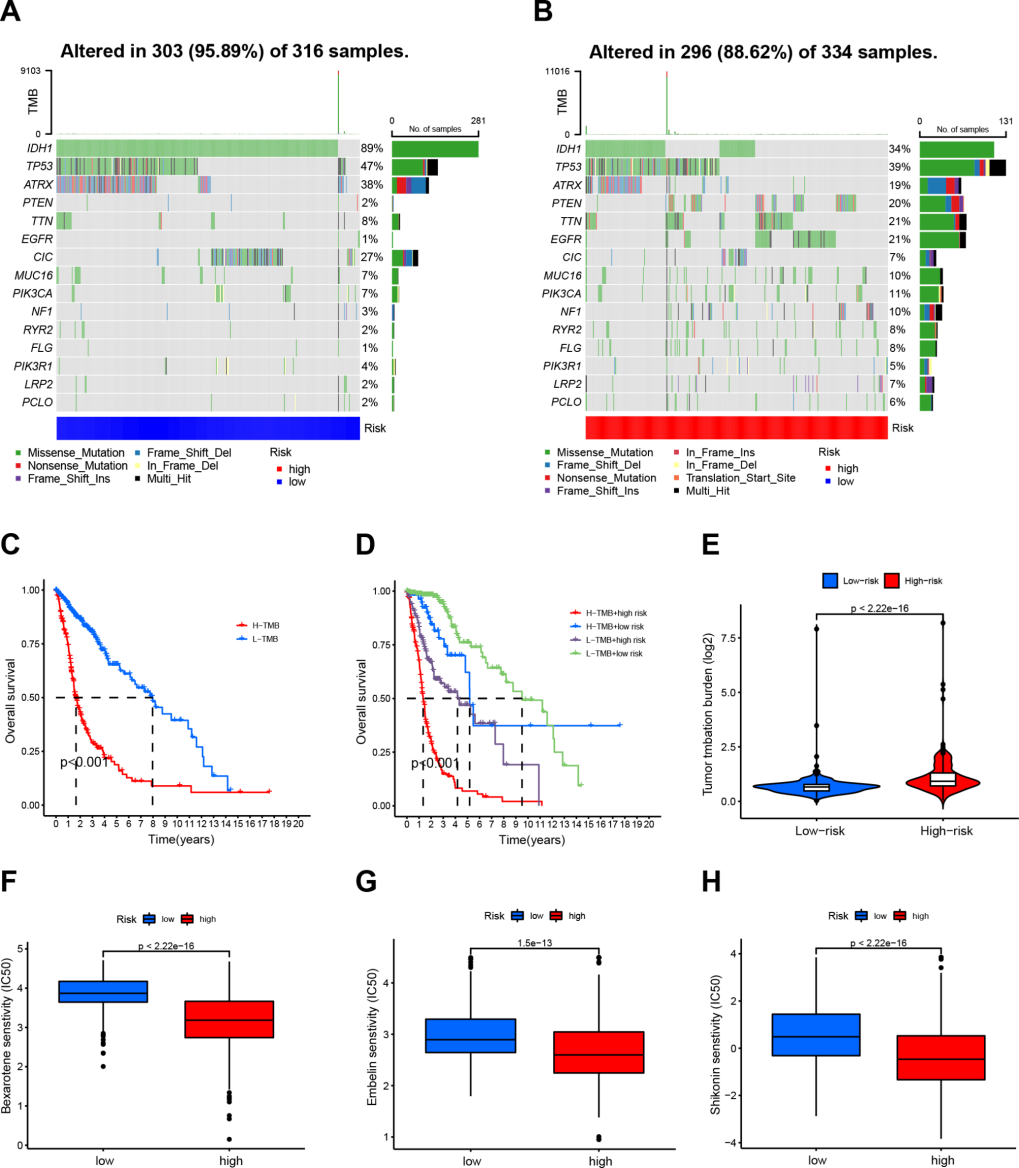
TMB analyses and drug sensitivity between high- and low-risk groups. (**A**-**B**) Waterfall plots displaying the mutation rates of the top 15 mutated genes of the low- (**A**) and high-risk groups (**B**). (**C**). The K-M curves of glioma patients in the TMB level-high and -low groups. (**D**). The K-M curves of the four subgroups based on the risk score and TMB levels. (**E**). The difference in TMB between high- and low-risk groups. (**F**-**H**). The box plot exhibiting drug sensitivity of bexarotene, embelin and shilkonin between high- and low-risk groups

We conducted a thorough examination of TMB-specific genes in both high-risk and low-risk groups. Our results suggested that low-risk individuals were more likely to have mutations in IDH1 and TP53, while high-risk individuals had mutations in PTEN, TTN, and EGFR (Fig. 8A and B). Patients were categorized into high- and low-TMB groups based on their TMB scores. Notably, there was a marked increase in OS for individuals with low TMB as compared to those with high TMB (Fig. 8C). We also evaluated the combined effects of TMB and DRL-scores on prognostic stratification, with high-risk patients having a poor prognosis regardless of their TMB status. Notably, the worst survivors were high-risk patients with high TMB, while the best survivors were low-risk patients with low TMB (Fig. 8D). The high-risk group scored higher on TMB than the low-risk group (Fig. 8E). Then, we evaluated the correlation between predictive characteristics and tumor immune-related drugs. The results revealed that bexarotene, embelin, and shikonin had lower IC50s in the high-risk group (Fig. 8F-H and S3C-E), which can help to detect individualized treatment regimens suitable for glioma patients.

### Consensus cluster analysis based on prognostic DRLs

**Fig. 9:**
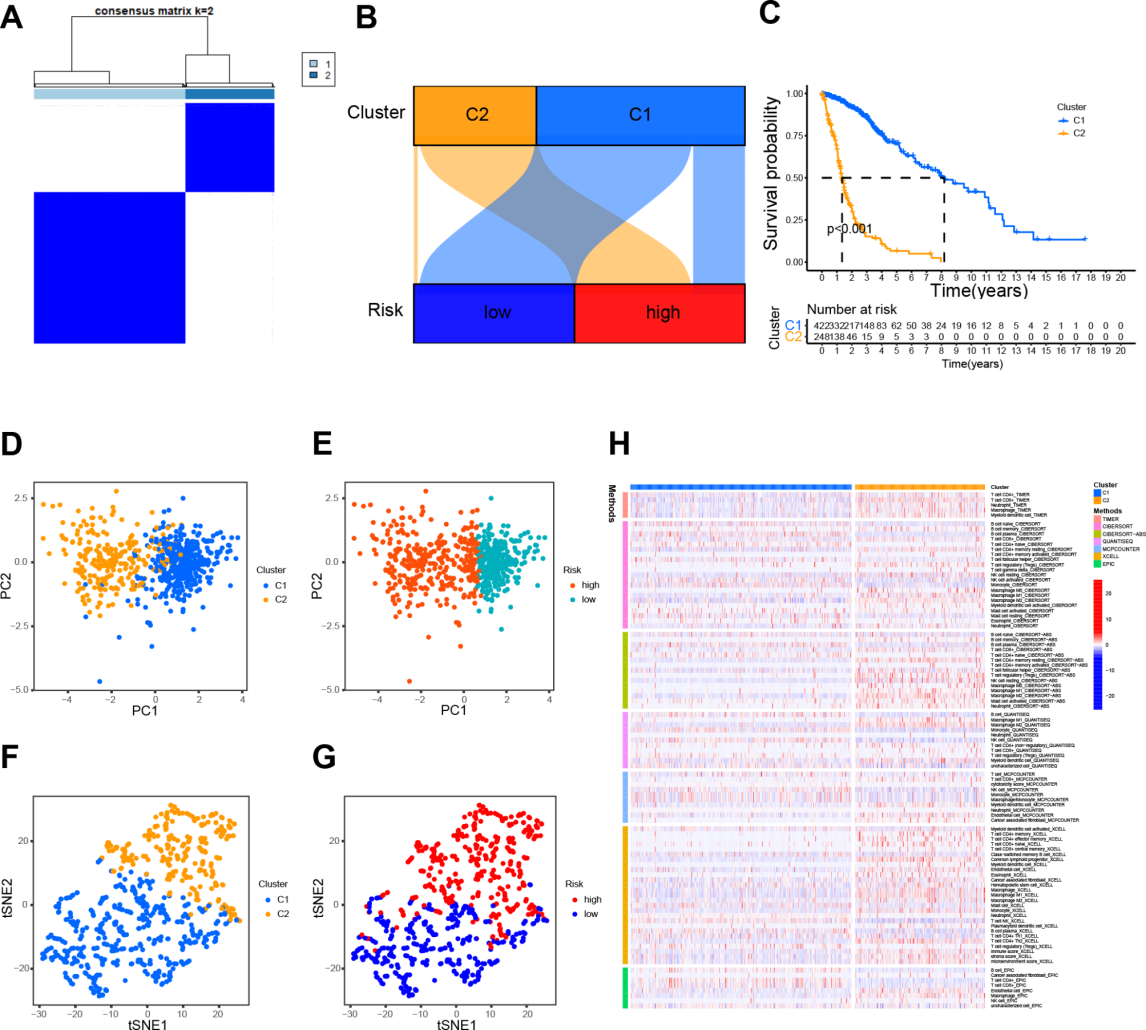
Consensus clustering analysis of DRLs and immune correlation analysis. (**A**). The consensus clustering matrix for k = 2. (**B**). A Sankey diagram showing the interaction between clusters and risk. (**C**). The Kaplan-Meier curves for overall survival of clusters 1 and 2. (**D**, **E**). Dimensional plots of PCA between the risk groups and clusters. (**F**, **G**). Dimensional plots of t-SNE between the risk groups and clusters. (**H**). The heatmap displaying the proportion of immune cells in the two clusters obtained by different algorithms

Next, we reclassified the 701 glioma specimens based on the DRL signature to investigate the immune landscape of different tumor subtypes. Using consensus cluster analysis, we segregated patients into two clusters (Fig. 9A-B). The patients in cluster 1 exhibited longer overall survival than those in cluster 2 (Fig. 9C). Principal component analysis effectively distinguished between the risk groups and clusters, as shown in Fig. 9D-E. The t-distributed stochastic neighbor embedding analysis confirmed the statistical equivalence between the two groups, as shown in Fig. 9F-G. We employed diverse algorithms to compute the proportion of immune cells across the two clusters and represented the dissimilarities between the two groups through heat maps, as shown in Fig. 9H. Accordingly, we found that cluster 2 had an abundance of immune checkpoint genes (Fig. S4A). It was found that cluster 2 had significantly higher scores for stroma, estimate, and immune function compared to cluster 1 (Fig. S4B-D), suggesting that the cluster 2 was associated with a stronger immune response and higher stromal content in the tumor microenvironment.

### Determining the potential clinical significance of DRLs

**Fig. 10:**
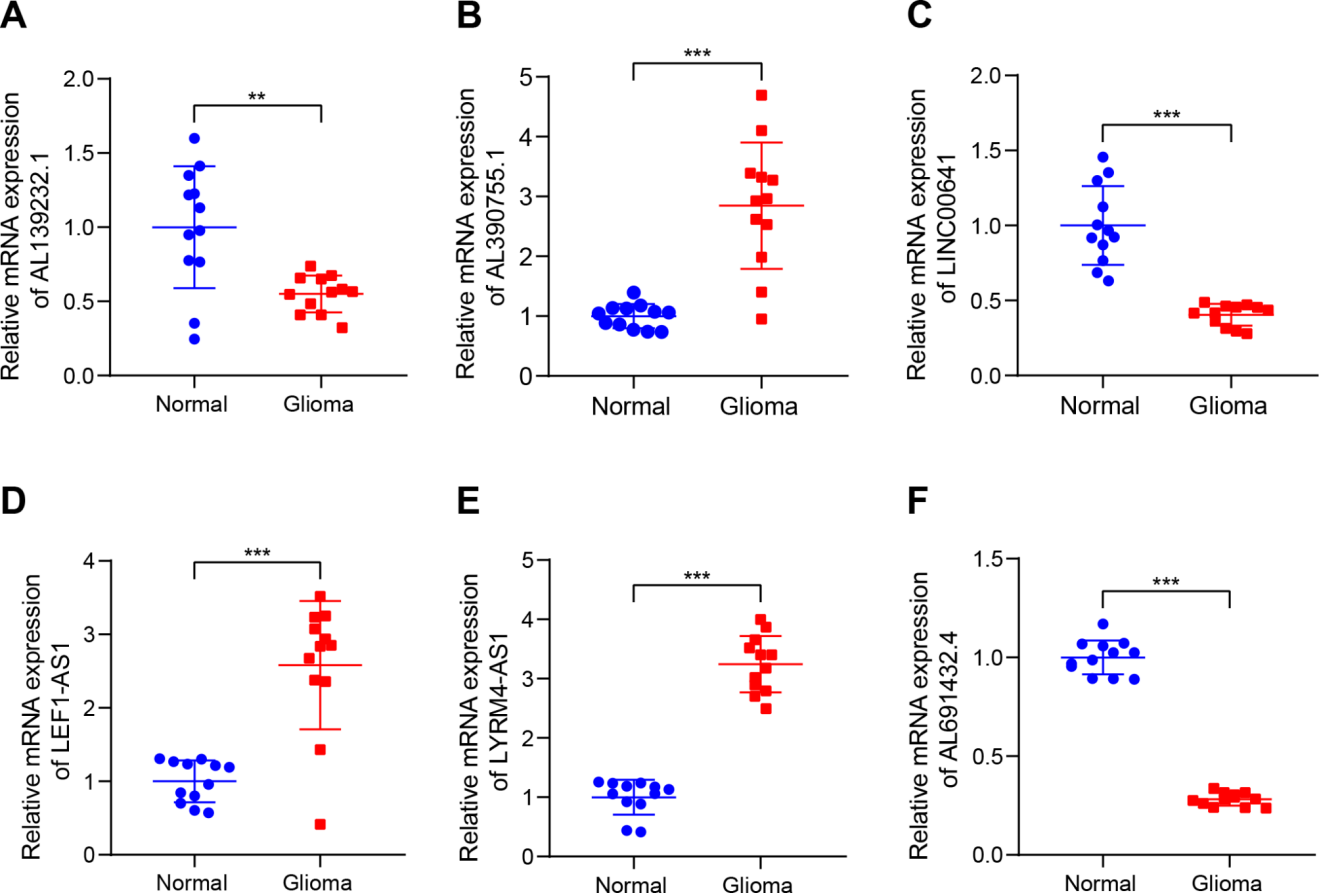
Validation of the expression levels of 6 DRLs in 12 non-tumor brain tissues and 12 glioma tissues. (**A**-**F**). The expression level of 6 DRLs (LINC00641, AL139232.1, AL390755.1, LEF1-AS1, LYRM4-AS1, and AL691432.4) in 12 non-tumor brain tissues and 12 glioma tissues. **p < 0.01, ***p < 0.001

To identify a prognostic biomarker associated with disulfidptosis related lincRNAs for glioma, we first observed the expression levels of six DRLs (LINC00641, AL139232.1, AL390755.1, LEF1-AS1, LYRM4-AS1, and AL691432.4) in glioma from the TCGA dataset. In glioma tissues, we found low levels of expression for LINC00641, AL139232.1, and AL691432.4, while high levels of expression were observed for AL390755.1, LEF1-AS1, and LYRM4-AS1 (Fig. S5A-F). Furthermore, by using RT-qPCR, we explored the expression of these six DRLs in glioma tissues and normal brain tissue, and our results matched those reported by the TCGA (Fig. 10A-F). High expression levels of LINC00641, AL139232.1, and AL691432.4 were significantly associated with longer overall survival (Fig. S5G, H, L), whereas low expression levels of AL390755.1, LEF1-AS1, and LYRM4-AS1 were also significantly associated with longer overall survival (Fig. S5I, J, K). Furthermore, we generated AUC values for the DRLs and observed that all DRLs had excellent discriminatory power for distinguishing patients with glioma (Fig. S5M-R).

## Discussion

Gliomas are neoplasms originating from the glial cells of the brain and represent a frequent subtype of primary brain tumors, bearing a high mortality rate and unfavorable prognoses [35]. Despite considerable progress in the treatment of glioma, its substantial inter- and intra-tumor heterogeneity poses significant challenges in identifying optimal therapeutic targets and treatment strategies [36]. Therefore, identifying innovative biomarkers for prognostication and prediction of therapeutic response holds paramount clinical significance for individuals afflicted with glioma.

An escalating number of novel modalities of non-apoptotic regulated deaths are being unearthed, and delving into more intricate mechanisms may lead to the revelation of novel therapeutic targets for cancer [37]. Disulfidptosis, an innovative type of programmed cell death, is presently undergoing intensive scrutiny in the field of tumorigenesis and therapies [6]. Various studies indicate that biomarkers related to disulfidptosis have substantial predictive power for cancer prognosis and treatment efficacy [6, 16]. Given the burgeoning evidence that lncRNA exerts a pivotal regulatory role in glioma growth and progression [38], DRLs should be assessed for their prognostic significance in glioma patients.

In the current investigation, we initially identified 102 DRLs with prognostic significance from the TCGA dataset. Out of these, we selected 6 DRLs (LINC00641, AL139232.1, AL390775.1, LEF1-AS1, LYRM4-AS1, and AL691432.4) to create the prognostic signature of DRLs. In both training and test sets, DRLs were able to forecast glioma patient survival outcomes with high accuracy. Subsequently, we discovered that OS rates were higher in the low-risk group. The time-dependent ROC curve analyses and calibration plots for the 1-year, 3-year, and 5-year survival projections substantiated the risk score’s superior predictive efficacy. Notably, the time-dependent concordance index analysis yielded values exceeding 0.70, thereby affirming the risk score’s robust prognostic capabilities. Intriguingly, both the AUC and concordance index results indicated that gender did not emerge as a statistically significant prognostic factor for glioma, performing even less effectively than a random probability benchmark of 0.5. Upon rigorous scrutiny, we speculated that an imbalanced gender distribution within the publicly accessible datasets and an insufficient overall sample size may be the underlying reasons for this. As well as comparing the stromal, ESTIMATE scores, and the abundance of immune cells within each risk subgroup, we evaluated the immune landscape differences. Ultimately, we validated the expression and diagnostic potential of the 6 DRLs in glioma by using both the TCGA database and our own clinical samples data.

To the best of our knowledge, a prognostic signature based on DRLs is not available yet. Here, we developed a prognostic signature with DRL consisting of six disulfidptosis-related lncRNAs (LINC00641, AL139232.1, AL390775.1, LEF1-AS1, LYRM4-AS1, and AL691432.4), some of which have been associated with tumor progression. For instance, LINC00641, located on human chromosome 14q11.2, has been demonstrated to have unique functional features and clinical significance in various cancers, including cervical cancer, renal cell carcinoma, and colorectal carcinoma [39–41]. Low levels of LINC00641 expression were observed in glioma cell lines, consistent with our findings, and it intervened in glioma growth by the miR-4262/NRGN pathway [42]. As noted previously, LEF1-AS1 expression was linked to poor prognosis of gliomas patients [9, 43]. Wang et al. showed that the LYRM4-AS1 protein is capable of affecting the growth of IL-1β-induced chondrocytes [44]. Those results are in line with those of our data mining. However, the roles and functions of AL139232.1, AL390775.1, and AL691432.4 have not been previously reported. In our study, we investigated disulfidptosis-related lncRNAs’ prognostic value in gliomas. Further laboratory testing validations at the molecular, cellular, and organismal levels are needed to investigate their potential effects on the disulfidptosis process.

Given the limited therapeutic options available to glioma patients, it is imperative to identify new targets for therapeutic intervention by comprehensively understanding the progression of gliomas in the brain tumor microenvironment. It is the unique immune mechanism of gliomas that determines a tumor’s microenvironment [45]. According to our findings, different risk subgroups exhibited DEGs that were related to the multiple immune-related biological processes, including leukocyte-mediated immunity, positive regulation of leukocyte activation, and B cell-mediated immunity. Furthermore, our study also found that high-risk group had elevated expression of immune checkpoint-related genes, such as CD44 and CD276. There is a poor prognosis with CD44 overexpression in gliomas, particularly those at WHO stage II and III [46]. CD44 has also been identified as a novel biomarker for M2 tumor-associated macrophages in glioma patients with a poor prognosis [47]. CD276 (B7-H3), a member of the B7 family of cell surface receptors, has been found to be an adverse prognostic factor for the patients with glioma [48]. Additionally, KEGG analyses revealed that these DRGs were enriched in several pathways including ECM-receptor interaction. It has been found that ECM receptors seem to be necessary for glioma cell migration, which can drive glioma cell migration and invasion [49]. Hence, targeting glioma-specific disulfidptosis pathways, including some DRLs, in combination with immunotherapy may offer a promising therapeutic approach for glioma patients.

Cumulative evidence suggests that the TMB is predicated on the number of somatic mutations and is associated with neoantigens that trigger antitumor immunity [50]. Our findings indicate that the low-risk group exhibited a higher mutation frequency than the high-risk group among the top 15 genes with the highest mutation rates. IDH1, TP53, ATRX, and CIC exhibited higher mutation frequencies in the low-risk group. ATRX deletions/mutations have been found to occur frequently in conjunction with IDH1 mutations and TP53 mutations [51]. It has been demonstrated that ATRX deletion leads to genetically unstable tumors that are more sensitive to double-stranded DNA damaging agents, thereby enhancing overall survival [52]. CIC mutations are rare in astrocytomas but have a higher likelihood of occurring in oligodendrogliomas and are linked to a lower malignant degree, according to radiomics and radiogenomics analysis conducted by Zhang’s laboratory [53]. These published findings are in agreement with our observations of higher survival rates in low-TMB groups.

Gathering literature underscores the significance of assessing drug sensitivity for therapeutic agents in determining tumor treatment efficacy [34]. Bexarotene treatment in C6 glioma cells impedes NF-κB activation by amplifying PPARγ expression, leading to the regulation of apoptosis, DNA damage, and ROS production, among other processes, consequently exerting anti-proliferative and cytotoxic effects [54]. Embelin has been reported to trigger glioma cell apoptosis through the mitochondrial pathway and inhibition of NF-κB activity [55, 56]. Shikonin, one of the primary efficacious components of Lithospermum erythrorhizon, causes apoptosis through several pathways [57, 58]. Shikonin also prompts necrosis by increasing the expression of RIP1 and RIP3 and promoting the formation of the RIP1/RIP3 necrosome [59]. Our research indicates that these three drugs exhibit significantly lower IC50 values in patients with high-risk glioma. Nonetheless, further studies on the disulfidptosis mechanism of these drugs warrant more scrutiny.

Undoubtedly, this study is not without limitations. Firstly, the prognostic signature was constructed based on publicly available datasets and, although validated using PCR expression assays, additional prospective experimental studies are necessary to validate its predictive capability. Furthermore, further studies with larger samples and different cohorts are required to enhance statistical power.

## Conclusions

In summary, our research has implications for predicting glioma patients’ prognosis as well as for clarifying the mechanism of lncRNA in disulfidiptosis of glioma. Meanwhile, our study may offer some useful insights for patients with glioma seeking novel immunotherapy and targeted therapy.

### Electronic supplementary material

Below is the link to the electronic supplementary material.

Supplementary Material 1

Supplementary Material 2

Supplementary Material 3

Supplementary Material 4

Supplementary Material 5

Supplementary Material 6

## Data Availability

Data from this study can be found in the TCGA-GBM and TCGA-LGG databases (http://cancergenome.nih.gov).

## Abbreviations

DRLs: Disulfidptosis-related lncRNAs
GO: Gene Ontology
KEGG: Kyoto Encyclopedia of Genes and Genomes
GSEA: Gene set enrichment analysis
ROC: Receiver operating characteristic
RT-qPCR: Reverse transcription quantitative PCR
TCGA: The Cancer Genome Atlas
LASSO: Least absolute selection operator
OS: Overall survival
PFS: progression-free survival
AUC: Area under the receiver operating characteristic
PCA: Principal component analysis
DEGs: Differentially expressed genes
TMB: Tumor mutation burden
