# Supplementary figures and images for: Leveraging a disulfidptosis‑related lncRNAs signature for predicting the prognosis and immunotherapy of glioma
Source: Cancer Cell Int. 2023 Dec 8;23:316. doi: 10.1186/s12935-023-03147-7 (PMC10709922; doi:10.1186/s12935-023-03147-7)

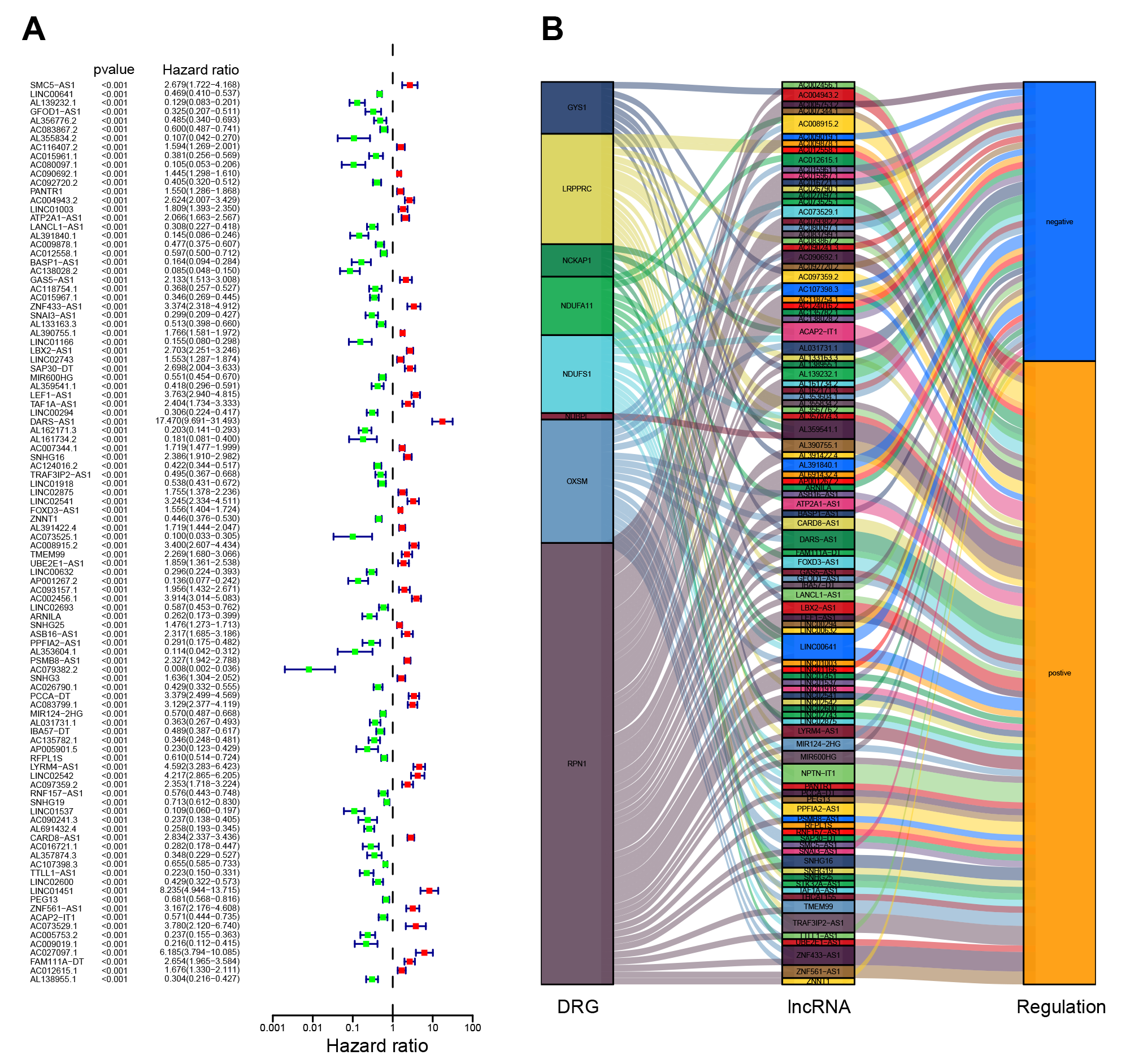

Supplement: Supplementary file 1 — Supplementary Material 1 [file 12935_2023_3147_MOESM1_ESM.png]

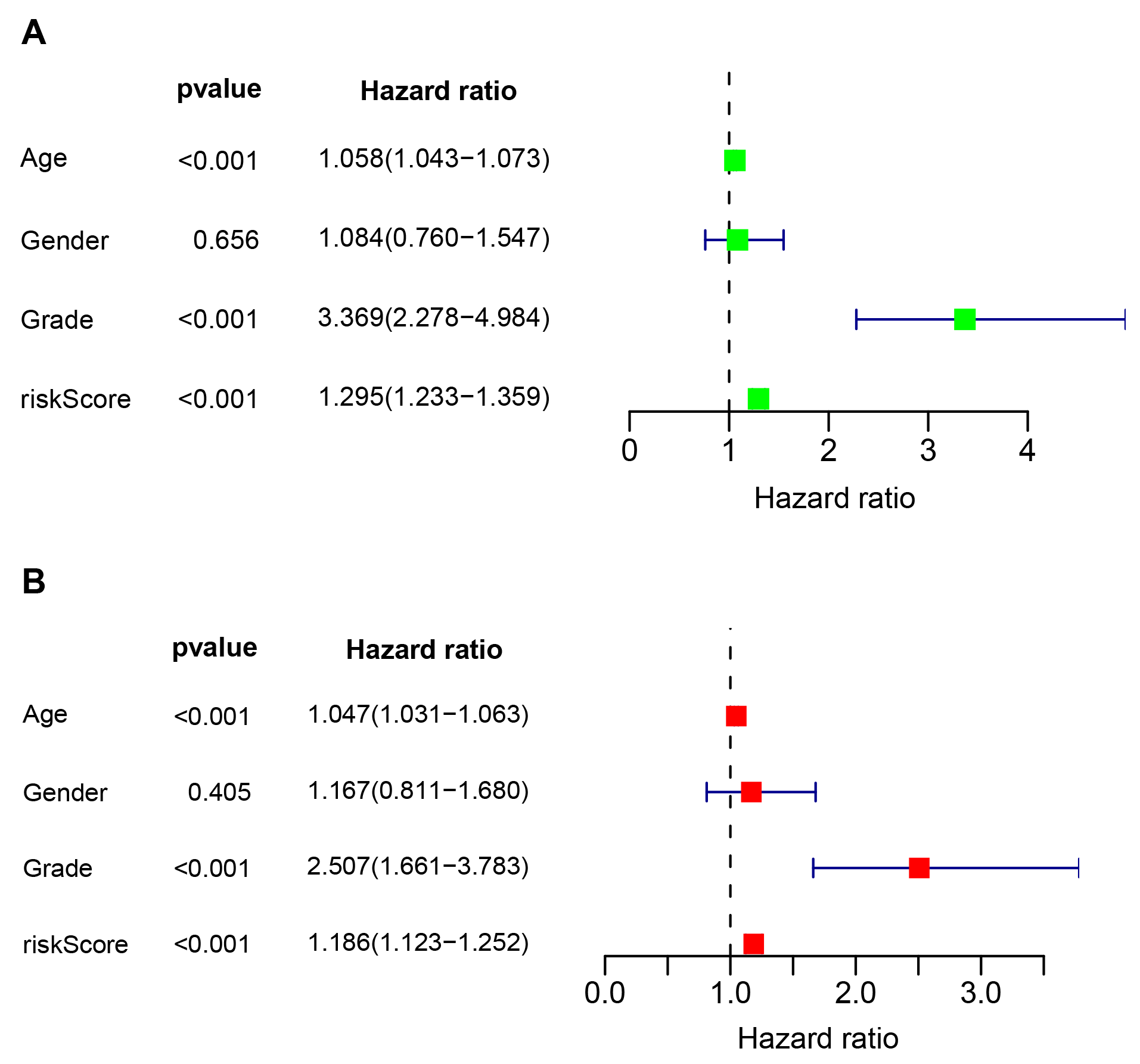

Supplement: Supplementary file 2 — Supplementary Material 2 [file 12935_2023_3147_MOESM2_ESM.png]

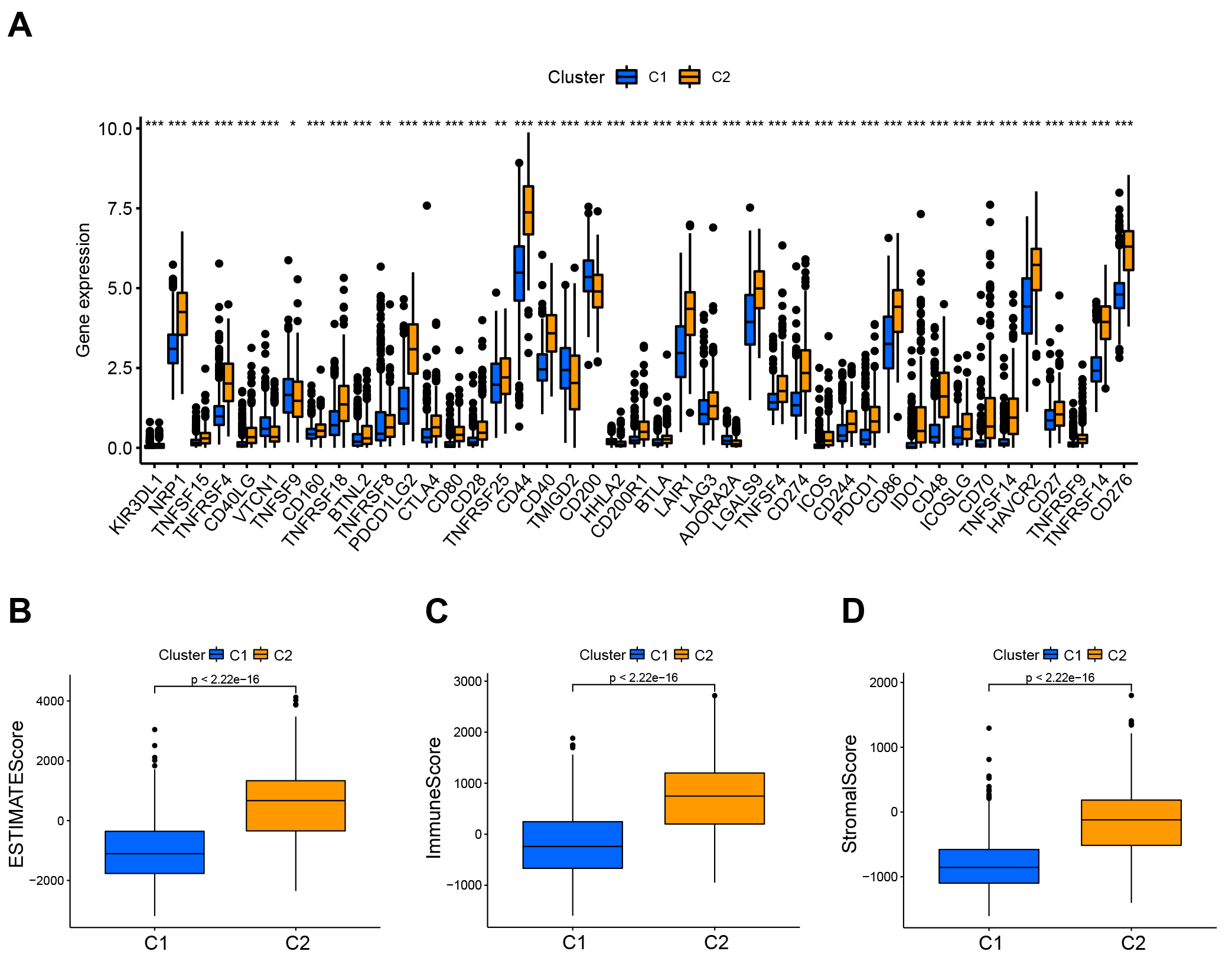

Supplement: Supplementary file 3 — Supplementary Material 3 [file 12935_2023_3147_MOESM3_ESM.png]

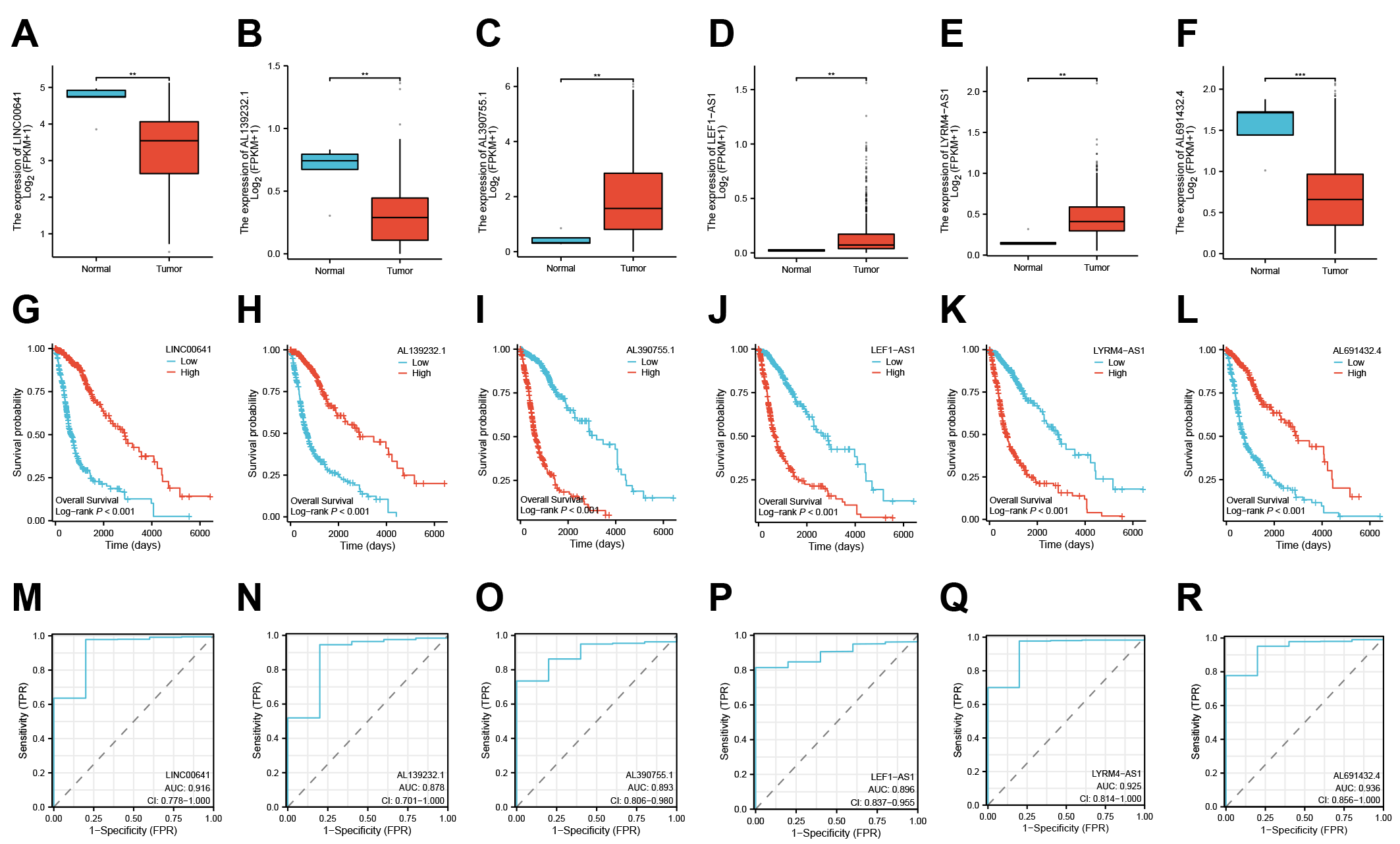

Supplement: Supplementary file 4 — Supplementary Material 4 [file 12935_2023_3147_MOESM4_ESM.png]

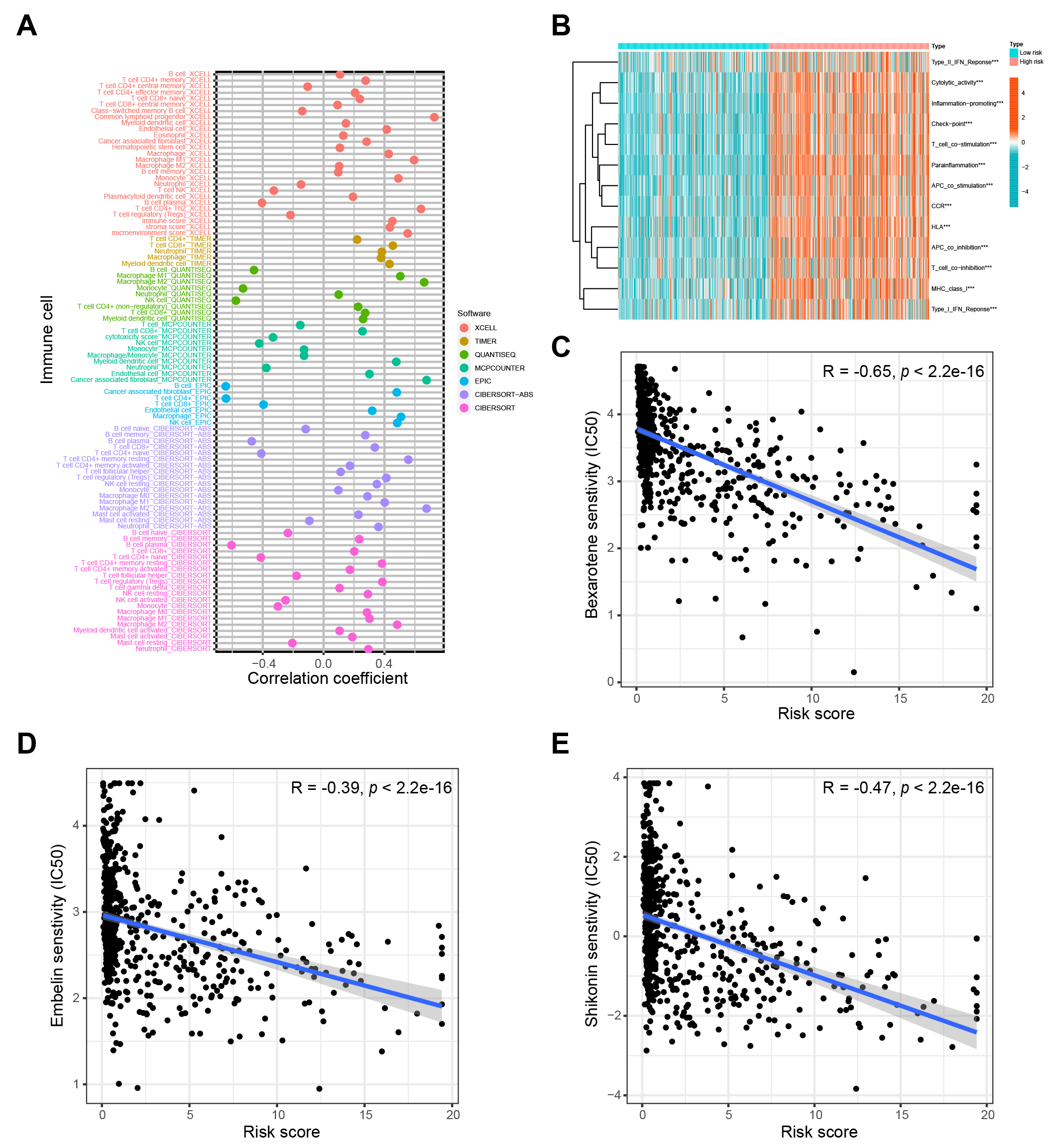

Supplement: Supplementary file 5 — Supplementary Material 5 [file 12935_2023_3147_MOESM5_ESM.png]
