# Supplementary material for: Leveraging a disulfidptosis‑related lncRNAs signature for predicting the prognosis and immunotherapy of glioma
Source: Cancer Cell Int. 2023 Dec 8;23:316. doi: 10.1186/s12935-023-03147-7 (PMC10709922; doi:10.1186/s12935-023-03147-7)
Supplement: Supplementary file 6 — Supplementary Material 6 [file 12935_2023_3147_MOESM6_ESM.docx]

**Supplementary figure legends**

**Supplementary Figure 1. Identification of prognostic CRLs and the correlation between DRLs and DRGs.**

(A). Forest plot of univariate analysis results showing OS-related DRLs. (B). Sankey diagram showing the interaction between DRGs and OS-related DRLs.

**Supplementary Figure 2. An independent prognostic analysis of clinical parameters and risk scores.**

(A). Univariate Cox regression analysis of the clinical characteristics and risk score with the OS. (B). Multivariate analysis of the clinical characteristics and risk score with the OS.

**Supplementary Figure 3. The immune infiltration in the high- and low-risk groups.** (A). A heatmap showing the immune infiltration in high- and low-risk groups using various algorithms (xCELL, TIMER, quanTIseq, MCP-counter, EPIC, CIBERSORT-ABS, and CIBERSORT algorithms). (B). The heatmap for the functions of immune cell subpopulations between the high- and low-risk groups. (C)-(E). The correlation between the risk score of DRLs signature and sensitivity of drugs such as bexarotene (C), embelin (D) and shilkonin (E).

**Supplementary Figure 4. The immune correlation analysis of different consensus clusters.**

(A). The box plot exhibiting the expression level of immune checkpoint genes between different clusters. (B-D). The ESTIMATE score, immune score, and stromal score in different clusters. *p < 0.05, **p < 0.01, ***p < 0.001.

**Supplementary Figure 5. Validation the potential clinic significance of 6 DRLs.**

(A-F). Box plots showing the expression levels of 6 CRLs used to establish the prognostic signature in gliomas using TCGA dataset. (G-L). Kaplan–Meier curves for overall survival in glioma patients with different expression of 6 DRLs. (M-R). Receiver operating characteristic (ROC) analysis of 6 DRLs expression showing a high ability to discriminate glioma tissues from normal samples using the data of TCGA.

**Supplementary Table S1: 10 disulfidptosis-related genes.**

| GYS1 | LRPPRC | NCKAP1 | NDUFA11 | NDUFS1 |
| --- | --- | --- | --- | --- |
| NUBPL | OXSM | RPN1 | SLC3A2 | SLC7A11 |

**Supplementary Table S2: Primer list of RT-qPCR.**

| Gene Name | Forward primer | Reverse primer |
| --- | --- | --- |
| GAPDH | GGAGCGAGATCCCTCCAAAAT | GGCTGTTGTCATACTTCTCATGG |
| LINC00641 | CACTTTTGCAGACCCTCACA | ACTTGACGGGTGGATTCTTG |
| AL139232.1 | CAGTTTTCCTGCCTCCGAGA | GAGCTCGGTACCCCTTCCC |
| AL390755.1 | GGA​AAG​CTA​TGA​GGA​AGA​AGA​AAC​AGA | CAA​CCT​GTG​CTG​TGA​TGA​ATG​G |
| LEF1-AS1 | CTACCCATCCTCACTGTCAGTC | GGATGTTCCTGTTTGACCTGAGG |
| LYRM4-AS1 | CCCAACTGTAACGACCAC | TTAACATCAAACTAAGGCAC |
| AL691432.4 | GAGAGACAGTGACCAAGCCC | GGTCCAAGTCAGTGCGGTAA |
